# Blast Overpressure Waves Induce Transient Anxiety and Regional Changes in Cerebral Glucose Metabolism and Delayed Hyperarousal in Rats

**DOI:** 10.3389/fneur.2015.00132

**Published:** 2015-06-17

**Authors:** Hibah O. Awwad, Larry P. Gonzalez, Paul Tompkins, Megan Lerner, Daniel J. Brackett, Vibhudutta Awasthi, Kelly M. Standifer

**Affiliations:** ^1^Department of Pharmaceutical Sciences, College of Pharmacy, University of Oklahoma Health Sciences Center, Oklahoma City, OK, USA; ^2^Oklahoma Center for Neuroscience, College of Medicine, University of Oklahoma Health Sciences Center, Oklahoma City, OK, USA; ^3^Department of Psychiatry and Behavioral Sciences, College of Medicine, University of Oklahoma Health Sciences Center, Oklahoma City, OK, USA; ^4^Department of Neurosurgery, College of Medicine, University of Oklahoma Health Sciences Center, Oklahoma City, OK, USA; ^5^Department of Surgery, College of Medicine, University of Oklahoma Health Sciences Center, Oklahoma City, OK, USA; ^6^Oklahoma City VA Medical Center, Oklahoma City, OK, USA; ^7^Department of Cell Biology, College of Medicine, University of Oklahoma Health Sciences Center, Oklahoma City, OK, USA

**Keywords:** blast injury, traumatic brain injury, anxiety, fluorodeoxyglucose, positron emission tomography imaging, glucose metabolism, acoustic startle response

## Abstract

Physiological alterations, anxiety, and cognitive disorders are strongly associated with blast-induced traumatic brain injury (blast TBI), and are common symptoms in service personnel exposed to blasts. Since 2006, 25,000–30,000 new TBI cases are diagnosed annually in U.S. Service members; increasing evidence confirms that primary blast exposure causes diffuse axonal injury and is often accompanied by altered behavioral outcomes. Behavioral and acute metabolic effects resulting from blast to the head in the absence of thoracic contributions from the periphery were examined, following a single blast wave directed to the head of male Sprague-Dawley rats protected by a lead shield over the torso. An 80 psi head blast produced cognitive deficits that were detected in working memory. Blast TBI rats displayed increased anxiety as determined by elevated plus maze at day 9 post-blast compared to sham rats; blast TBI rats spent significantly more time than the sham controls in the closed arms (*p* < 0.05; *n* = 8–11). Interestingly, anxiety symptoms were absent at days 22 and 48 post-blast. Instead, blast TBI rats displayed increased rearing behavior at day 48 post-blast compared to sham rats. Blast TBI rats also exhibited suppressed acoustic startle responses, but similar pre-pulse inhibition at day 15 post-blast compared to sham rats. Acute physiological alterations in cerebral glucose metabolism were determined by positron emission tomography 1 and 9 days post-blast using ^18^F-fluorodeoxyglucose (^18^F-FDG). Global glucose uptake in blast TBI rat brains increased at day 1 post-blast (*p* < 0.05; *n* = 4–6) and returned to sham levels by day 9. Our results indicate a transient increase in cerebral metabolism following a blast injury. Markers for reactive astrogliosis and neuronal damage were noted by immunoblotting motor cortex tissue from day 10 post-blast in blast TBI rats compared to sham controls (*p* < 0.05; *n* = 5–6).

## Introduction

The direct effects and mechanisms of blast overpressure waves that cause a mild traumatic brain injury (blast TBI) are still not clearly understood, partly due to the complex nature of blast exposures and partly due to the psychological and physical polytrauma associated with the blast (1–3). The factor most unique to blast TBI compared to non-blast closed head TBI (such as sports injuries and car accidents) is the presence of an overpressure wave component. Blast-induced TBI is the most common injury of modern warfare, and has been titled as its “signature wound” (4). The nature of modern warfare and the frequent use of improvised explosive devices (IEDs) have led to increases in mild to moderate TBIs, but the availability of advanced protective gear and medical care at the battlefield have enabled military personnel to survive from otherwise lethal blast injuries (Defense and Veterans Brain Injury Center’s website: http://dvbic.dcoe.mil/). Although the exact numbers of U.S. service members suffering from blast TBI are not known, studies estimate that 60–80% of military TBI cases were caused by blast exposures (5).

Clinical evaluations of soldiers with TBI have detected neuronal and cognitive deficits 1–2 years after a blast incident (5, 6), highlighting the long-lasting consequences of blast TBI on the quality of lives for both service members and their families. Common symptoms experienced by blast TBI patients are memory deficits and concentration problems, chronic headache, dizziness, excessive fatigue and irritability, as well as vestibular, sleep, and visual disturbances (7–12). Service members exposed to blasts are more prone to chronic conditions such as major depression, generalized anxiety disorder (GAD), and post-traumatic stress disorder (PTSD). Coexisting diagnoses of blast TBI and one or more of those chronic conditions in patients have been linked to increased morbidity (13–22). Many of these anxiety, depression, and attention deficit disorders have been attributed to hyperarousal, a change (decrease or increase) in startle responses, and decreases in acoustic sensorimotor gating (23).

Cerebral glucose metabolism correlates significantly with neuronal function and activity of the injured brain (24–26). Studies on animal models of non-blast TBI confirm metabolic alterations within the brain that vary between hypometabolism to hypermetabolism depending on the severity of TBI, the model used, and the time of metabolic measurements relative to the TBI incident (24, 25, 27–32). Clinical positron emission tomography (PET) imaging studies using ^18^F-FDG have detected glucose hypometabolism in selected brain regions. These studies were done in cohorts of U.S. service members exposed to repeated or single blast TBI that occurred on average, 3–3.5 years prior to the imaging (33, 34). The acute cerebral glucose metabolic status of an injured brain following a single blast TBI has never been reported. This study provides PET imaging data that address cerebral glucose demand at 1 and 9 days following a single blast exposure that will help fill this gap in information.

The purpose of the current study was to determine the behavioral, physiological, and acute biochemical parameters associated with a single blast exposure using a previously validated blast TBI model (35, 36). Those studies extensively characterized physiological effects of the unshielded blast TBI [representative of a moderate-to-severe TBI with 40–60% mortality rate (35)]; as well as the head-directed blast with a shielded-torso [representative of a mild TBI with no mortality] (36). The head-directed blast with the shielded torso produced mild TBI with cognitive deficits and neuroinflammation, making it a useful model to understand CNS-mediated effects of head-directed blast-induced mild TBI. Therefore, we examined the effect of a single *head-only* overpressure blast injury (with no peripheral body exposure) on working memory, anxiety, cerebral glucose metabolism, acoustic startle response (ASR), and sensorimotor gating in Sprague-Dawley male rats in order to determine the contribution of brain-specific responses to blast TBI.

## Materials and Methods

### Animal care

Male Sprague-Dawley rats (*n* = 76; 225–250 g) were purchased from Charles River Laboratories International Inc. (Wilmington, MA, USA). Animals were housed in pairs with *ad libitum* access to food and water and a 12 h dark/light cycle. Rats were allowed to acclimate for 1 week to recover from transportation. All protocols were approved by the University of Oklahoma Health Sciences Center (OUHSC) Institutional Animal Care and Use Committee and the USAMRMC ACURO animal care committee.

### Blast-induced traumatic brain injury

**Figure 1:**
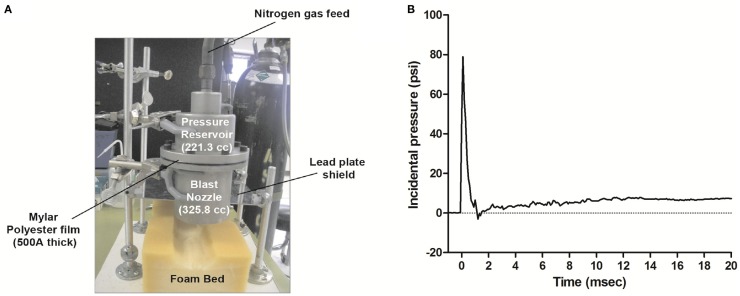
**Blast wave generation and detection**. Blast waves were produced using the generator apparatus pictured in **(A)**. The blast generator nozzle was positioned over the head of the animal, and each rat was exposed to a single overpressure wave with the body shielded by a lead plate. Pressure values were recorded using a piezoelectric transducer (PCB Piezotronics Inc., NY, USA) every 0.1 ms. **(B)** Representative blast wave from a single blast illustrating an incident peak pressure value of 80 psi (rise time of 0.2 ms), duration of 1.47 ms, a positive impulse (AUC) of 31.5 psi/ms, and a negative pressure of −3 psi at 1.2 ms. The mechanics of the overpressure wave simulation by the Conwep software may be found in Table S1 in Supplementary Material.

Prior to blast, anesthesia was induced with 4% isoflurane/100% O_2_ for 4 min and maintained with 2.5% isoflurane/100% O_2_ for another 2 min. Rats were secured in the supine position to a foam pad to prevent concussive impacts with the table below and to minimize acceleration/deceleration rotation of the head. The animal was positioned with the blast wave generator nozzle centered directly over the head (device shown in Figure 1A). The chest was shielded from the blast by a 2 mm thick lead plate to prevent damage to air filled organs such as lungs, gut, and kidney that could contribute to peripheral blast TBI mechanisms. The anesthesia nose mask was removed prior to initiation of the blast. Personnel were protected against the noise of the blast (maximum 100 dB) during the administration of the blast pressure wave with protective ear-muff guards (NRF 20). Rats were randomly group-matched into sham or blast groups based on their weight on the day of the blast, such that average weights of groups were similar. Sham rats received anesthesia and were restrained without blast exposure, whereas the blast group was exposed to either a single blast of 80 psi incidental pressure as measured by the pressure signal transducer (Model: S112A21, PCB Piezoelectonics Inc., NY, USA) using DASY lab v. 9.0 software (National Instruments, TX, USA). This pressure was chosen based on previous calibration studies that showed this overpressure exposure to induce mild TBI histopathology and behavioral deficits (35, 36). The time it took for rats to awaken and adjust to an upright posture after blast and/or anesthesia was noted for each rat as the recovery time. For pressure assessment, the signal transducer was placed in the exact same position where the rat’s head normally would be located (1 cm from nozzle edge and in center of nozzle’s diameter). Calibration curves were performed per manufacturer’s instructions for converting volt values recorded by the transducer into psi pressure values. Transducer sensitivity was 42.94 mV/psi. The intensity of peak incident pressure values were controlled by changing the distance of the transducer from the blast nozzle in increments of 1 cm. Blast wave calibration measurements indicated linearity of pressure–distance proportions. Blast wave parameters based on simulations from Conwep software may be found in Table S1 in Supplementary Material.

### Experimental design

After receiving either sham or blast TBI, the behavioral and imaging studies were performed as follows: in one set of rats, working memory (beginning day 1 post-blast) was measured as well as anxiety at days 22 and 48 (8/group). In a second set of rats, PET imaging was performed on days 1 and 9 post-blast (5–6/group). In a third set of rats, anxiety symptoms were assessed at day 9 post-blast (10–11/group); vestibulomotor studies (days 1–8 post-blast from that same set of rats are in review; Awwad et al.). In the last set of rats, ASRs were determined at day 15 post-blast (7–8/group) from the same rats that generated learning and memory data published in Tompkins et al. (36).

### Morris water maze apparatus

For the Morris water maze (MWM) protocols listed below, rats were tested in a large circular water maze (6 ft diameter) with a water level of 30 cm at 25 ± 1°C. The top half of the water tank was painted black, and the water was mixed with non-toxic black tempera paint. The circular platform, also referred to as the island, was 10 cm in diameter and its height was adjusted above or below the water according to the protocol used below. Rat navigation was monitored and recorded using the video-tracking ANY-maze software (Stoelting Inc., IL, USA).

### Matching-to-sample spatial working memory MWM paradigm

Sixteen rats (8/group) were tested in the MWM with the platform hidden 2 cm below the surface of the water in the presence of spatial cues as indicated below. Each rat received two identical trials to find the hidden platform in the MWM according to the protocol by Vorhees and Williams (37). The sample trial (trial 1) consists of moving the platform every day to a new location, whereas the location of the platform in the matching trial (trial 2) is identical to trial 1 each day for a total of 21 days (e.g., the acquisition phase). Trial 1 is a measure of learning the new platform location, while trial 2 is a measure of working memory. If the rats remember the location of the platform from trial 1 based on the spatial cues and their working memory is intact, then the rat performance time on trial 2 will be less than the performance time on trial 1. After the acquisition phase, the rats underwent a random phase for 6 days where the platform is moved daily for trial 1 and moved again to a new location for trial 2. This phase eliminates any non-spatial strategies that the rats may have developed in the previous acquisition phase. The test phase, also known as the reinstatement phase, was conducted in the same manner as the acquisition phase to show that rats use spatial working memory to find the platform. Trials ended after 120 s or upon finding the platform; inter-trial intervals were 15 s. If rats did not locate the platform, they were manually guided to the platform. The data are presented as a percent ratio of the difference between trials 1 and 2 during each block of 3-day intervals to initial trial 1 during the acquisition phase for that group.

### Cued navigation MWM paradigm

Cued navigation MWM was used as an inclusion criterion in all the MWM studies to rule out any possible blast-induced deficits in swimming motor function and/or vision. None of the blast animals were excluded from the MWM studies based on this metric. Rats were tested on the indicated days following the MWM trial for that day (*n* = 8/group). For this assessment, the platform was clearly visible, extending 1 cm above the surface of the water and topped with a circular white foam board. Cued navigation was further enforced using a white flag made of foam board that extended 7 cm above the platform via a copper wire. Spatial cues on the walls were removed to ensure that each rat located the platform solely by visual observation in a hippocampal-independent manner, from four different entry points. Each attempt to reach the visible platform from a different entry point was considered one trial. Every rat had four trials, and the location of the platform was changed to a different quadrant in every trial. Trials ended after 120 s or upon finding the platform. Cued navigation was monitored and recorded as latency to reach the platform using the video-tracking software ANY-maze (Stoelting Inc., NY, USA). Each animal was allowed to remain on the platform for 30 s after reaching the platform, then tested for the next trial after a 30 s interval.

### Elevated plus maze method

Anxiety was evaluated using the elevated plus maze (EPM) on days 9, 22, and 48 post-blast in two separate sets of sham and 80 psi head blast groups as described above (*n* = 8–11/group), according to the protocol described by Walf and Frye (38). Briefly, rats were placed in the center zone (junction of the open and closed arms) of the EPM. Rats were allowed to explore the plus maze arms for 5 min, during which the average time the rat spent per visit in the open arms, the number of times the rats showed rearing behavior, and the total distance traveled were recorded and tracked using the video-tracking software ANY-maze (Stoelting Inc.). The time that the rats spent in either open or closed arm is represented as the fraction of time in arms (total time − time in center zone = total time in arms).

### Acoustic startle response and pre-pulse inhibition

Acoustic startle response and pre-pulse inhibition (PPI) studies were performed to assess the integrity of sensorimotor function in relation to anxiety 15 days following sham or blast injury. For measurement of ASRs, rats were placed in non-restraining rat holders inside the sound attenuating Acoustic Startle Chambers (Med Associates). Rat holders were each cleaned with a mild soap solution between each animal session. A continuous 70 dB background white noise was presented throughout the experimental session. Following a 5 min period for acclimation to the chamber, the animals received a series of trials in which acoustic startle stimuli (50 ms bursts of white noise) were presented and startle responses were recorded. Trials were separated by inter-trial intervals that varied randomly between 10 and 30 s, with an average inter-trial interval of 20 s. The first three trials consisted of presentations of 110 dB stimuli to allow the establishment of a stable baseline response; these trials were not included in the data analysis. Rats were then exposed to acoustic stimuli of 0, 100, 105, 110, and 115 dB, presented in pseudo-random order such that the intensity of each stimulus was presented 10 times during the next 50 trials. PPI was determined during the final 20 trials, as 115 dB stimuli were presented alone or preceded by a 90 dB pre-pulse (20 ms duration). During each of the experimental trials, behavior was recorded during a 100 ms baseline period, a 50 ms period following the presentation of the pre-pulse (either 0 or 90 dB), and a 100 ms startle response period following presentation of the acoustic stimulus. Movement during the baseline period was used to establish a threshold for detection of startle responses, where the threshold was equal to the maximum movement plus four times the SD of baseline movement. ASRs were calculated as the peak-to-peak amplitude between the maximum and minimum movement detected during the 100 ms period following the acoustic stimulus and were included in subsequent analyses if this exceeded the threshold. Responses that did not exceed the threshold were discarded unless all of the responses for stimulus intensity were below threshold, and then a score of 0 was assigned and was included in the analyses. ASR data were analyzed using the SAS GLM analysis (equivalent to the two-way repeated ANOVA; *n* = 7–8) and PPI data were analyzed using a one-way ANOVA (*n* = 5–6) to determine the significance of group differences in the response to varying acoustic stimulus intensities and in PPI, respectively.

### Positron emission tomography and computerized tomography scans

All PET and computerized tomography (CT) imaging were performed at the Research Imaging Facility (University of Oklahoma Health Sciences Center, College of Pharmacy). The same amount of radiotracer ^18^F-fluorodeoxyglucose (^18^F-FDG; 100 μCi/rat) was intravenously injected into each animal, as all animals were similar in weight.

### Glucose metabolism by ^18^F-FDG

As a glucose analog, ^18^F-FDG is utilized by the brain in a fashion similar to that of glucose. Therefore, glucose metabolism can be quantified directly by the uptake of ^18^F-FDG into the brain. Clinical grade ^18^F-FDG was obtained from IBA Molecular (Dallas, TX, USA). Rats were food-deprived overnight; ^18^F-FDG was administered intravenously via tail vein to both sham and blast rats and allowed to distribute in the body for 45 min. Rats were imaged for the next 45 min under 2.5–3% isoflurane anesthesia using the X-PET/CT machine (Gamma Medica-Ideas, Northridge, CA, USA). Plasma samples were collected immediately before and after imaging; ^18^F-FDG levels in each sample were determined immediately after collection in a well γ-counter. Plasma samples also were assayed for glucose concentration using the quantitative colorimetric Quantichrom™ Glucose Assay kit – DIGL-200 (BioAssay Systems, Hayward, CA, USA). The imaging procedure was repeated on day 9, followed immediately by euthanasia. Rats were euthanized by induction of anesthesia at 4–5% isoflurane and exsanguinated by intracardiac blood withdrawal. Brains were excised, flash-frozen, and stored at −80°C for further biochemical analysis. PET imaging was performed in a separate group of rats than those used in behavioral studies.

### PET image analysis

The acquired image data were reconstructed using a filtered back projection algorithm. A CT scan was acquired for each rat following PET imaging to establish anatomic landmarks. Both PET and CT were fused together using Amira v.3.1 software (Visage Imaging Inc., San Diego, CA, USA) provided with the imaging system. The accumulation of ^18^F-FDG was estimated by the region of interest (ROI) method using the volume of the entire brain as one region, as described earlier (39). The Standard uptake value (SUV) was calculated using the equation: SUV = [(B_c_/Pl_c_ × Pl_glu_)/D], where B_c_ represents counts detected by PET per minute of imaging per volume of the brain, Pl_c_ represents the radioactive counts per minute per volume of plasma, Pl_glu_ represents the glucose content in milligrams per milliliter of plasma, and D represents the i.v. dose of the PET tracer administered in μCi (40). PET/CT fused images captured by Amira v.3.1 software were overlaid with the Rat Brain Atlas using the Brain Navigator software (41, 42). The skull outline from CT images was used as a guide for the Bregma position and for determining distances from Bregma (in millimeters) for overlays with the Atlas images.

### Immunoblotting

Frozen brains from rats used in the day 9 EPM paradigm and euthanized on day 10 post-blast were thawed and sliced into 2 mm thick sections using a coronal brain slicer (Zivic Instruments, PA, USA). Slices were immersed in ice-cold Kreb’s buffer and hippocampi and cortices dissected. After homogenization, tissues were lysed with cell lysis buffer (50 mM Tris, 0.05 M NaCl, 50 mM NaF, 10 mM EDTA, 2 mM EGTA, 1% Triton-X 100, pH 7.4) containing Complete^®^ protease inhibitor cocktail tablet (Roche Applied Science, Indianapolis, IN, USA). Supernatants (14,000 × *g* at 4°C for 10 min) were measured for protein, solubilized in Laemmli buffer, and heated to 65°C for 15 min. Samples (40 μg) were resolved by SDS-PAGE, transferred to PVDF membranes, and probed for the following proteins: amyloid precursor protein (APP) (ab15272, Abcam; 1:500), caspase 3 (#9665, Cell Signaling; 1:1000), and glial fibrillary acidic protein (GFAP) (RB-087-R7, Thermoscientific Inc.; 1:200). Immunoreactive (IR) bands were visualized by chemiluminescence (Pierce ECL substrate) and captured with the UltraLum Omega Imaging System (UltraLum, CA, USA); densitometric analysis was performed using Ultra Quant 6.0 software. Band density was normalized to the loading control actin.

### Statistical analysis

Data were analyzed using the appropriate statistical test using Graphpad Prism v. 5 or SAS program as indicated.

## Results

### Blast injury model: Direct 80 Psi head blast without peripheral/systemic involvement

To further understand the mechanisms and effects of blast wave TBI to the brain specifically and its response to the blast, blast overpressure waves were directed only to the head as described in methods. Based on calibration curves and measurements, rats subjected to a single blast were exposed to peak incidental pressure values around 80 pounds per square inch (psi). The blast loading mechanics of our blast overpressure wave were quantified and the following parameters are reported as recommended by Sundramurthy et al. (43): peak incidental pressure 77.2 ± 3.5 psi with a time to rise of 0.167 ± 0.03 ms, the duration of the blast wave was 1.4 ± 0.05 ms, and a positive impulse (area under the positive overpressure phase) of 30.5 ± 1.18 psi/ms (*n* = 3) (Figure 1B).

Anesthetized Sprague-Dawley male rats were exposed to sham injury (anesthesia only) or a single 80 psi overpressure loading directed to the head. All the rats survived the blast TBI and rats in all groups had similar righting reflex recovery times (data not shown; Awwad et al. manuscript in review), consistent with the absence of an immediate systemic (vagal) response to the blast (44). There were no significant differences in size or weight between the groups at any time point after the blast. Brains from blast TBI rats did not show any contusions or signs of hemorrhage upon gross anatomical external examination of the brains, and were similar to sham rats in appearance.

### Blast TBI-induced working memory deficits

**Figure 2:**
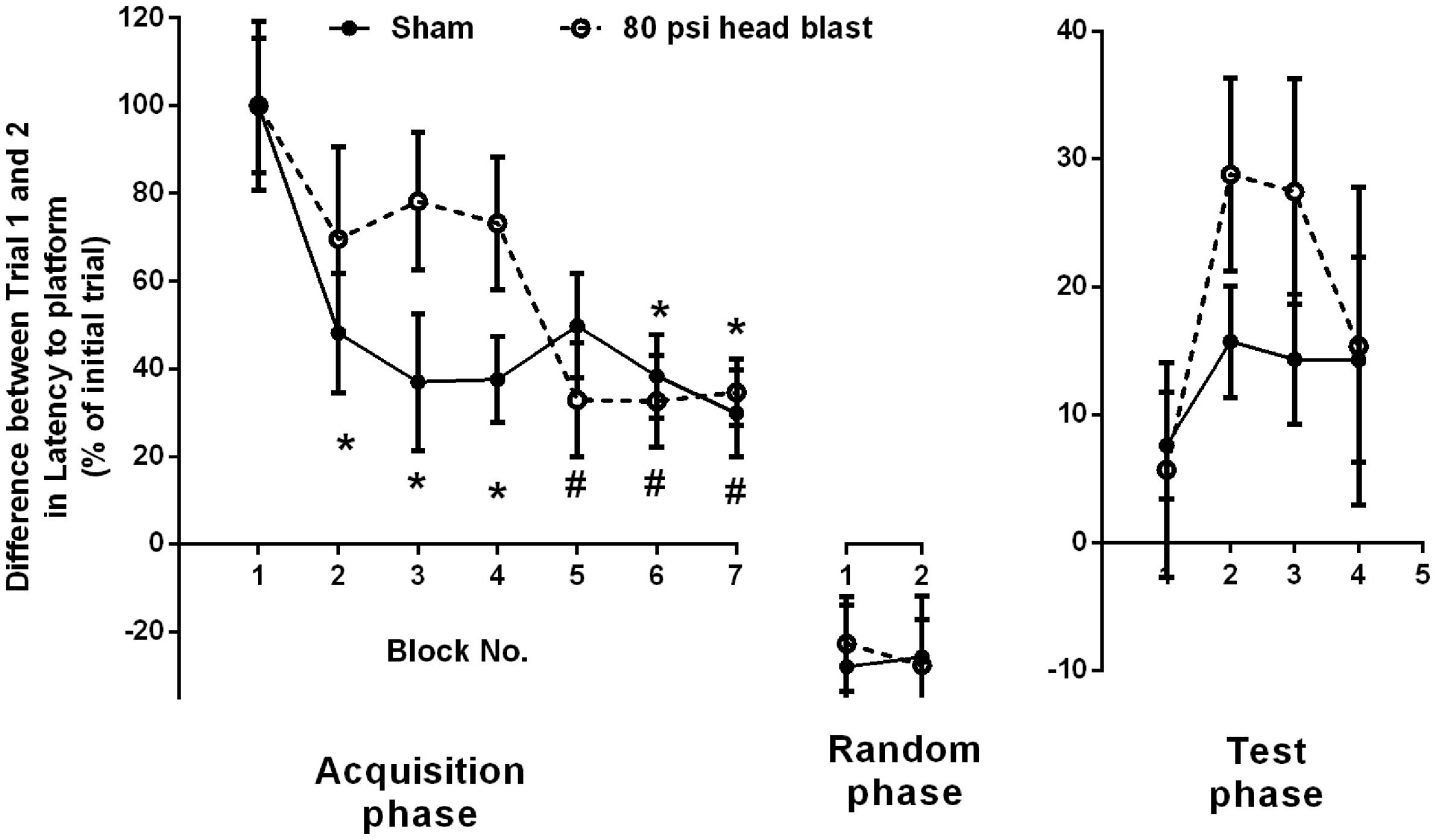
**Blast exposure impairs working memory performance measured by MTS-MWM**. Blast TBI rats failed to exhibit improvements in learning in the second trial of the acquisition phase [as indicated by higher ratio difference between the sample trial (Trial 1) and matching trial (Trial 2) compared to sham until block 5 (day 13-15; significantly reduced)], whereas sham rats showed significant reduction as early as block 2. Two-way repeated measure ANOVA, Bonferroni’s *post hoc* analysis *p < 0.05 vs. block 1 in Sham group, and ^#^*p* < 0.05 vs. block 1 in 80 psi head blast (*n* = 8/group). Data are presented as mean ± SEM. Data analysis from both the acquisition and testing phases was performed within each group and between groups for the latency to find the platform in the MTS-MWM paradigm. No differences between groups were noted in the random phase.

Rats exposed to head-directed overpressure waves were evaluated for cognitive deficits in their working memory using MWM (37). The effect of 80 psi blast TBI on the working memory paradigm known as matching-to-sample (MTS)-MWM (37) was tested. Briefly, Trial 1 is a measure of learning the task to search for a new platform location in a different quadrant. Trial 2 is a measure of working memory for finding the location of the platform found in Trial 1. The difference between trials was calculated as “Trial 1–Trial 2” and presented as a percent of the first Trial 1 in the first block per group for latency to reach the platform (Figure 2). Data are presented in blocks of 3-day intervals that began day 1 post-blast. While sham rats displayed a change in their trial difference ratios as early as block 2, performance of blast TBI rats did not improve until block 5; 80 psi head blast TBI trial difference ratios were similar over the first 12 days post-blast (blocks 1–4), indicating an impairment in working memory. The sham rats were able to learn and use their working memory by the end of 3 days, whereas the 80 psi head blast rats took over 12 days to learn and use their working memory efficiently. During the random phase, both groups performed similarly, with similar trial difference percent ratios. Interestingly, 80 psi head blast TBI rats took longer (Figure 2) and traveled further (data not shown) than the sham group to reach the platform in the test phase (the third section of the MTS-MWM) for blocks 2 and 3, yet did not reach statistical significance. Two-way repeated measures ANOVA performed for acquisition and test phases show a significant effect of time [*F*_(9,414)_: latency = 9.47 and distance = 9.29, *p* < 0.0001] but not blast exposure [*F*_(1,46)_: latency = 0.5962 and distance = 0.6487 for blast *p* > 0.05], and no significant interaction between blast exposure and time [*F*_(9,414)_: latency = 1.10 and distance = 1.18, *p* > 0.05]. Bonferroni’s *post hoc* multiple comparison analysis indicates a significant difference between block 1 and every other block in sham rats, whereas 80 psi head blast TBI rats show a significant difference between block 1 and blocks 5 through the last block. There were no significant differences between individual time point comparisons between blast TBI and sham rats group.

Therefore, rats exposed to 80 psi head blast showed impaired working memory compared to sham controls as measured in the MTS-MWM paradigm (Figure 2). Average swimming speeds across trials and between groups did not vary and therefore could not have contributed to the differences in cognitive function between the groups (data not shown). Performance in the cued navigation MWM paradigm was used as an inclusion criterion in all the MWM studies to rule out possible blast-induced contributions to either motor swimming or visual deficits. None of the blast animals were excluded from MWM studies based on this metric. Motor function, as determined by swimming ability, was similar between sham and blast rats on day 1 (Sham: *n* = 48, 18.4 ± 3.0 s; 80 psi head blast: *n* = 48, 13.8 ± 1.6 s), by Student’s *t*-test (*p* = 0.139), and day 8 (Sham: *n* = 32, 30.8 ± 4.0 s; 80 psi head blast: *n* = 28, 24.9 ± 4.8 s, *p* = 0.352).

### Blast-TBI induces transient anxiety and delayed hyperarousal

**Figure 3:**
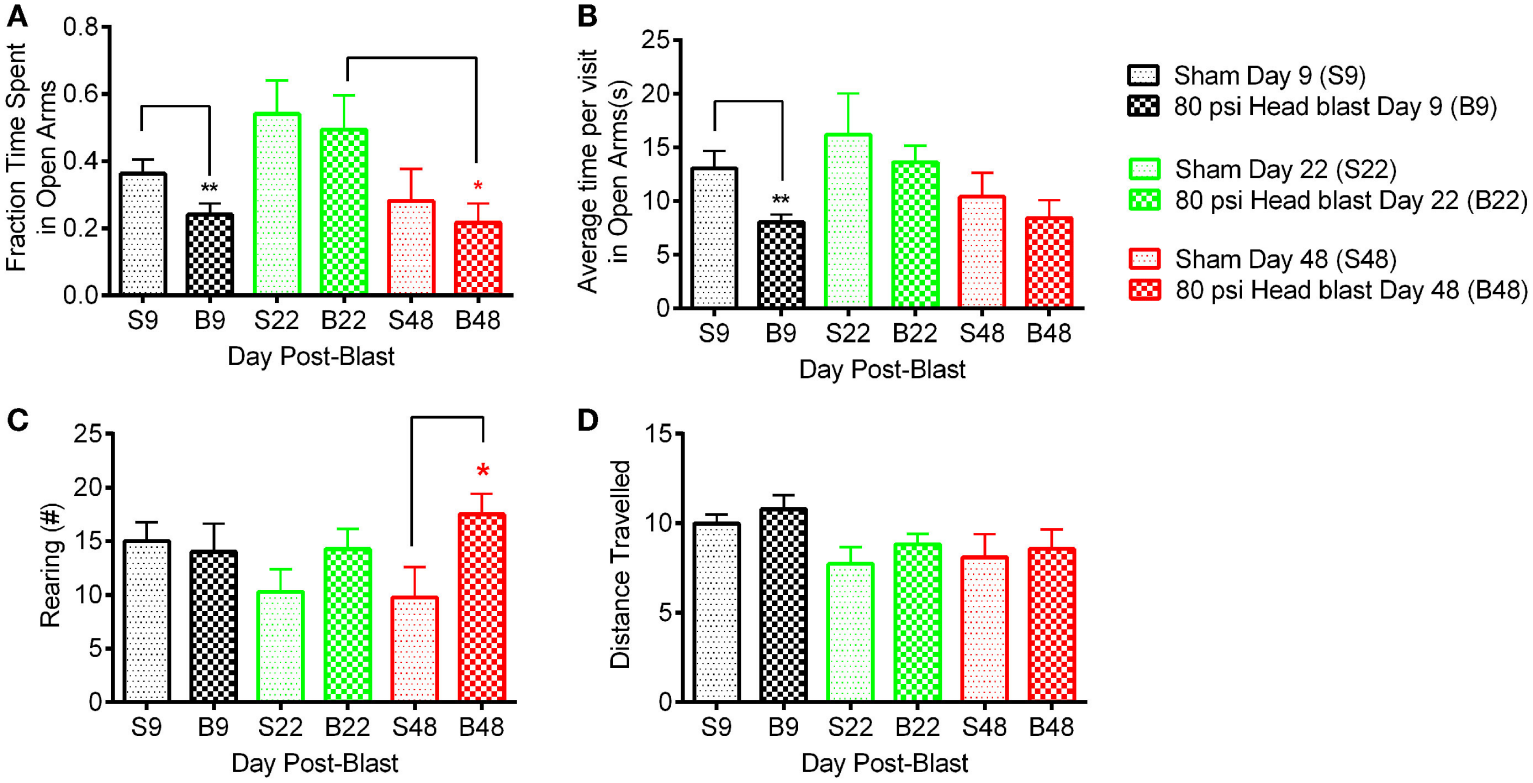
**Blast-exposed rats exhibit anxiety-like behavior 9 days post-blast and hyper-arousal 48 days post-blast**. A total of 80 psi head blast rats tested on day 9 spent significantly less time in the open arms **(A)** and less average time in each visit to the open arms **(B)** compared to the sham rats (un-paired Student’s *t*-test: **p < 0.01, *n* = 11 blast and 10 sham). Hyper-arousal was noted in 80 psi head blast rats, which exhibited excessive rearing compared to sham rats **(C)** on day 48 post-blast compared to sham rats (two-way repeated measures ANOVA with Bonferroni *post hoc* analysis: *p < 0.05, *n* = 8/group). Rat mobility/motor function was not affected by the blast overpressure wave as both groups traveled similar distances **(D)** on days 9, 22, and days 48 post-blast/sham. Data are presented as mean ± SEM.

Assessment of anxiety symptoms was determined using the EPM (38) on day 9, or days 22 and 48 post-blast. Rats exposed to 80 psi head blast displayed signs of anxiety 9 days post-blast compared to sham rats, as shown by the increased fraction of time spent in closed arms, decreased fraction of time spent in open arms (Figure 3A), and reduced mean time spent in open arms per visit (Figure 3B). Differences detected on day 9 post-blast did not result from reduced mobility in blast-exposed rats as distance traveled per rat did not differ between groups (Figure 3D). Data analysis of anxiety symptoms in blast TBI and sham rats at day 9 used an unpaired Student’s *t*-test, since that was the only time point assessed in this set of rats (fraction time in open arms: *p* = 0.033, average time per visit in open arms: *p* = 0.009, rearing number: *p* = 0.771, and distance traveled: *p* = 0.411). EPM was measured in a different group of rats at 22 and 48 days post-blast.

In the second set of rats comparing day 22 and day 48, no differences in fraction of time spent in open arms, average time per visit in the open arm, and distance traveled were noted between the 80 psi head blast and sham rats (Figures 3A,B). Two-way repeated measures ANOVA indicated a significant effect of time for fraction of time spent in open arms as well as average time per visit in the open arms [*F*_(1,14)_: fraction time in open arms = 12.38; *p* = 0.0034 and average time per visit in open arms = 8.54; *p* = 0.011], but not blast exposure [*F*_(1,14)_: fraction time in open arms = 0.28; *p* = 0.6039 and average time per visit in open arms = 0.41; *p* = 0.534]; no significant interaction between time and blast exposure was found [*F*_(1,14)_: fraction time in open arms = 0.011; *p* = 0.916 and average time per visit in open arms = 0.0096; *p* = 0.923]. Bonferroni’s *post hoc* comparison analysis indicates a significant difference in the fraction of time spent in open arms between day 22 and 48 for the 80 psi blast rats (*p* < 0.05), but no significant differences within sham or between sham and 80 psi head blast groups. No differences in the *post hoc* comparisons for either the average time per visit in open arms or the distance traveled parameters were noted.

Excessive rearing, associated with hyper-arousal and anxiety, was noted in blast TBI rats on day 48 compared to sham rats and to both groups on day 22 (Figure 3C). Two-way repeated measures ANOVA indicated a significant effect of 80 psi head blast TBI on the number of rearings [*F*_(1,14)_ = 6.449; *p* = 0.0236], but no significant effect of time [*F*_(1,14)_ = 0.1447; *p* = 0.7094] or significant interaction between blast exposure and time [*F*_(1,14)_ = 1.06; *p* = 0.3194]. Excessive rearing is consistent with increased emotional alertness or hyper-arousal, not hyperlocomotion (45–47), since the 80 psi head blast rats traveled similar distances as the sham rats (Figure 3D).

### Blast TBI and alterations in cerebral glucose metabolism

**Figure 4:**
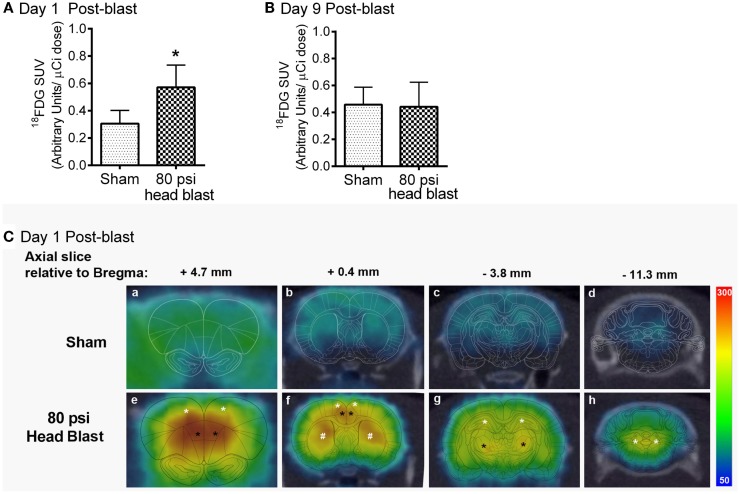
**Transient increase in cerebral glucose metabolism was noted in the brain 1 day, but not 9 days, after an 80 psi head blast**. ^18^F-FDG (radioactive glucose analog) SUVs in the whole brain following a 100 μCi i.v. dose were increased 1 day after blast TBI **(A)** compared to that in sham rats, but no differences were noted by day 9 **(B)** post-blast (Student’s unpaired *t*-test: *p < 0.05: *n* = 4–6/group). Data are presented as mean ± SEM **(C)**. Representative axial PET/CT fused images of ^18^F-FDG uptake within the brain of a sham (a–d) and a blast (e–h) rat on day 1 post-blast. Images were overlaid with the corresponding rat brain atlas map as indicated in reference to distance from Bregma using Brain Navigator software (41). Images represented from a sham rat and an 80 psi head blast indicate that regions with highest intensity of ^18^F-FDG uptake (red > yellow > green > blue) were found in motor cortex (white * in e and f), frontal orbital cortex (black * in e and f), caudate putamen (white * in f), hippocampus (white * in g), thalamus (black * in g), and vestibular nuclei (white * in h).

To determine the effect of head-only blast TBI on acute cerebral glucose metabolism, glucose metabolism was semi-quantified by calculating the SUVs of ^18^F-FDG for the whole brain. Global cerebral glucose metabolism was significantly increased on day 1 post-blast in the brains of the 80 psi head blast group compared to sham rats (Figure 4A), but returned to levels similar to those of sham rats at day 9 post-blast (Figure 4B). PET/CT fused images representing the mean SUV values from a representative rat are shown, with the rat brain atlas overlaid, using Brain Navigator software (41). Figure 4C highlights the significant regional increases on day 1 post-blast in brain regions associated with cognition, vestibulomotor and sensorimotor function, and anxiety. Areas most sensitive to primary blast exposure as indicated by increased ^18^F-FDG uptake 1 day post-blast include the prefrontal orbital cortex, parietal cortex (sensorimotor cortex), hippocampus, caudate-putamen, thalamus, and vestibular nuclei (Figure 4C). Axial, sagittal, and horizontal views representing cerebral glucose metabolism on days 1 and 9 in representative sham and blast rats may be seen in Figure S1 in Supplementary Material.

### Blast TBI-induced changes in acoustic startle response

**Figure 5:**
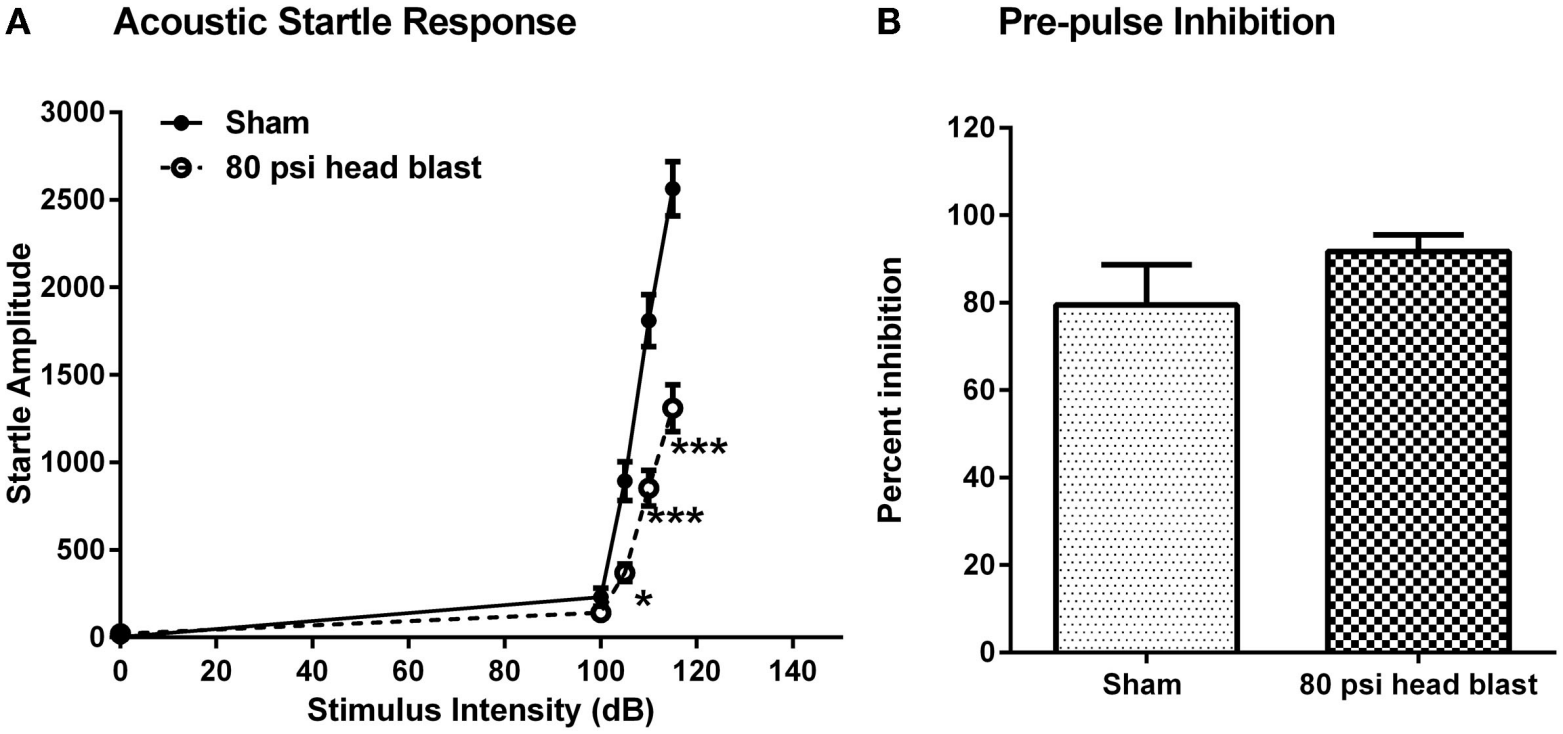
**Blast exposure reduces startle responses to acoustic stimuli but does not affect PPI 2 weeks post-injury**. **(A)**. Rats showed significantly lower responses to acoustic stimuli at 105, 110, and 115 dB upon randomized noise bursts of 0, 100, 105, 110, and 115 dB. Repeated measures two-way ANOVA: *p < 0.05 and ***p < 0.001, *n* = 7–8. **(B)**. There was no significant difference between blast and sham rats’ responses measured as inhibition produced by pre-pulse exposure to 90 dB followed by a 115 dB stimuli. ANOVA: *p* > 0.05. Data are presented as mean ± SEM.

Two weeks post-blast, blast-TBI rats exhibited significantly lower startle amplitude responses to acoustic stimuli (Figure 5) compared to sham rats, such that there was a significant interaction between group and stimulus intensity [SAS GLM *F*_(1,52)_; group = 8.37, *p* < 0.0001, and intensity = 72.95; *p* < 0.0001]. Rats also were subjected to PPI to measure the activation of sensorimotor gating at 115 dB in response to an acoustic pre-pulse of 90 dB. There was no difference between the groups in the inhibition produced by pre-pulse exposure [one-way ANOVA *F*_(1,9)_ = 1.18; *p* > 0.05], consistent with intact hearing in the 80 psi blast rats.

### Blast TBI and biochemical markers of injury, astrogliosis, and apoptosis

**Figure 6:**
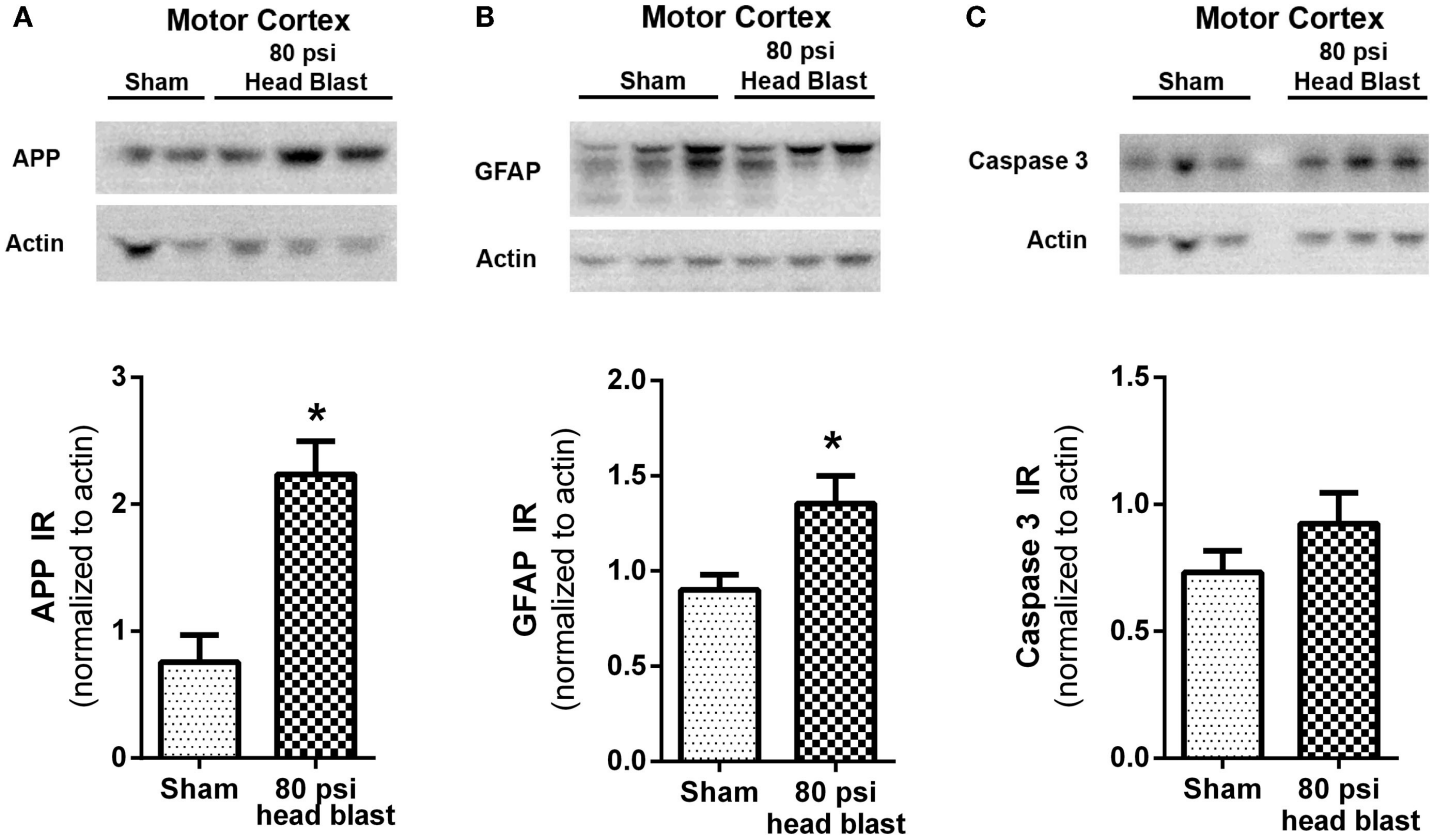
**Blast-induced increases in APP and GFAP are detected in the motor cortex**. The motor cortex region extracted from 80 psi head blast rats showed increased **(A)** APP (neuronal damage) and **(B)** GFAP (reactive astrogliosis) but not **(C)** caspase 3 (pro-apoptotic marker) immunoreactivity (IR) 10 days post-blast compared to sham rats. Samples were visualized by chemiluminescence as shown in the representative blots showing two to three rats per group and densitometric analysis is shown in the respective graphs. IR was normalized to actin as the loading control. Blast TBI rats were compared to sham rats using Student’s *t*-test (*p < 0.05; *n* = 5–6). Data are presented as mean ± SEM.

Expression of injury markers APP, GFAP, and caspase 3 from day 10 post-blast and sham rats was quantified by densitometric analysis of immunoblots from cortical (motor cortex) and subcortical tissue (hippocampus). GFAP and APP were increased in the motor cortex of blast rats (Figure 6; *p* < 0.05 by unpaired Student’s *t*-test; *n* = 5–6/group) compared to sham rats; no differences in caspase-3 expression were noted. No detectable changes in expression levels of APP, GFAP, and caspase 3 in the hippocampus of blast exposed rats compared to sham rats were noted at day 10 post-blast (data not shown).

## Discussion

A substantial body of preclinical data and neuroimaging evidence in patients confirms that primary blasts produce neuronal damage that translates into behavioral and neurological impairments in blast TBI (35, 45, 48–53). To develop a complete understanding of the causes and consequences of brain injury resulting from blast exposure, it is important to examine contributions resulting from the effects of blast to the head alone. Our blast model was previously validated as a means to examine the perspective of blast overpressure loading to the head alone in the absence of confounding peripheral contributions from external injuries and other complex components of a blast (35, 36).

Exposure to a single blast wave to the head using our model induces: (1) early deficits (1–2 days post-blast) in learning memory (36), (2) intermediate deficits such as anxiety (Figure 3) and impaired ASR (Figure 5) at 9 and 15 days post-blast respectively, and (3) long-term hyperarousal (Figure 3) and working memory deficits (Figure 2) that were detected several weeks post-blast. These findings imply that the exposure of the head to blast overpressure waves alone substantially contributes to the delayed cognitive deficits in tasks of executive function, attention, and decision-making, as those reported in military personnel with blast TBI usually detected 1–2 years after their deployment (54, 55). These findings are consistent with a recent study that reported long-term impairment in cognitive neurophysiology 4 weeks post-blast using electrophysiological recordings from mouse brain slices (56). We also detected anxiety symptoms followed by long-term hyperarousal in blast TBI rats; hallmark symptoms of PTSD (57), and insomnia (58, 59). Therefore, the model replicates many of the debilitating long-term deficits in executive function and memory performance, and psychological effects that are comorbid with blast TBI (10, 20–22).

One of our goals was to directly examine cerebral glucose utilization acutely and post-acutely in blast-TBI, since this question has not been addressed in the blast TBI literature. We report a blast-induced transient increase in cerebral glucose metabolism (hypermetabolic state) 1 day post-blast that is absent when rats are re-tested 9 days later. Glucose is the major substrate for metabolism in the brain and has been studied extensively in non-blast TBI. Cerebral glucose metabolism following brain injury is very dynamic and complex (varies by region); correlating with neuronal integrity, function, and synaptic activity (24–26, 60). Similar transient increases in cerebral glucose metabolism have been noted in both clinical and preclinical non-blast TBI studies (27–30, 32, 61), usually followed by reduced glucose metabolism that eventually returns to normal or below normal depending on the severity of the injury (62). This hypermetabolic state detected 1 day post-blast (Figure 6) correlates with a state of increased glycolysis similar to that reported in both clinical and preclinical TBI studies (9, 27–30, 32, 61, 63). PET/CT fused images allow us to visualize the presence of the hypermetabolic state in brain regions associated with executive (prefrontal orbital cortex, parietal cortex, hippocampus) and vestibulomotor (motor cortex, caudate putamen, thalamus, and vestibular nuclei) function. Hypermetabolism is usually triggered by an energy deficit eventually leading to ionic homeostatic disruptions with axolemmal damage/leakage (27, 28, 64). Over time post-TBI, glucose stores become depleted and the hypermetabolic state becomes exhausted, resulting in reduced cerebral glucose metabolism (9); hypometabolic state may develop if blood flow and energy demand remain uncoupled (28, 29). Cerebral glucose metabolism returned to levels seen in sham rats by day 9 post-blast. Though the ^18^F-FDG data do not provide evidence of hypometabolism at day 9 post-blast, parallel PET imaging studies that utilized a hypoxia marker did reveal accumulation of the hypoxia marker 8 days post-blast (Awwad et al. manuscript in revision). In contrast, a transient hypometabolic state was noted 1 day following fluid percussion brain injury that lasted for a couple of days; that hypometabolic state was further aggravated and sustained longer after a subsequent brain injury (31). Our study is the first to directly examine glucose uptake at acute (1 day) and post-acute (9 day) time points following blast TBI that is not as feasible on the battlefield. Of course, it is important that these metabolic studies be extended to include additional time points within and beyond the current points assessed, as well as expanded to include additional markers of hypoactivity to determine when long-term cerebral glucose hypometabolism previously reported in U.S. veterans 3–4 years after repeated blast TBI would become apparent in blast TBI rats (33).

Shear stress, mechanical stretching of the brain tissue/neurons, acceleration/deceleration of the brain within the intracranial space (1, 9, 56) increased intracranial pressure, cerebral vasospasm (65), and thoracic contributions from the periphery to the CNS (35, 44, 66–68) can all contribute to primary blast TBI. Additionally, mild TBI in general is commonly linked to disturbances in cerebral metabolism, hemodynamics, and ion homeostasis that ultimately lead to the release of oxygen radicals, axolemmal damage, uncoupling of cerebral blood flow (CBF), and metabolic demand (corresponding to increased glucose utilization) that subsequently results in neuronal degeneration and tissue damage via apoptosis (9, 28, 69–71). Potential triggers for the energy deficit linked to direct overpressure waves include cerebral vasospasms (8, 72, 73) changes in intracranial pressure, and reductions in CBF (71, 73–76). The uncoupling of CBF from glucose metabolic demand disrupts the homeostatic metabolic state causing an increased demand in metabolism (28), similar to that reported in this study (Figure 6). Another potential mechanism for the transient increase in cerebral glucose metabolism noted in this study is a brief disruption of the blood brain barrier (BBB) that has been demonstrated in blast and non-blast TBI models (77–79). BBB disruption in our blast model could explain the transient increase in ^18^F-FDG uptake on day 1 post-blast through excessive permeability across the BBB tight junctions and endothelia. The brain always attempts to restore BBB function in response to injury (80–82); normal glucose uptake levels on day 9 post-blast may reflect that BBB recovery. Future studies will determine whether increased glucose metabolism in this model (and others) is a result of blast-induced BBB disruption.

Blood brain barrier disruption also could explain the axonal damage and reactive astrogliosis noted in the cortical brain region (motor cortex) that exhibited biochemical changes in APP and GFAP compared to the subcortical brain region (hippocampus). Accumulation of the β-amyloid precursor protein is a marker of axonal damage in neurons induced by trauma (83, 84), and has been detected in blast TBI (52, 79). Astrocytes are activated almost immediately in response to a brain injury (astrogliosis), and are necessary for proper neuronal function (85). Astrogliosis, detected by increases in the expression of the GFAP, is a common response of the brain to blast TBI (2, 51, 79, 86, 87). Apoptotic cell death also is a common consequence of brain injury and contributes to functional deficits. Caspase 3 is one of the earliest proteins activated when apoptosis is triggered by either intrinsic or extrinsic pathways. Increases in cleaved caspase 3 and caspase 3 have been noted with blast TBI (52, 88, 89). Diffuse axonal injury (DAI) noted in associated brain regions (3, 6, 79) could ultimately contribute to long-term cognitive and anxiogenic disorders. Since the primary purpose of this study was not to assess the extent of DAI, we cannot comment on the presence or absence of DAI in our model other than the accumulation of APP in the motor cortex region is consistent with axonal damage in that part of the brain. Histopathological examination of H&E slides further confirm that this injury was a mild TBI as observed by lack of significant tissue damage or injury to the integrity of the tissue. No visible signs of damage or neurodegeneration were visible in the 80 psi head blast TBI rat brain compared to sham, similar to previous reports in other mild blast TBI models (90). The disturbance of metabolic homeostasis is a clear indication of direct biochemical change in brain tissue following blast exposure. Histopathological studies on blast injured rats using our blast model show an increase in neurodegenerative, neuroinflammatory, and injury markers as described previously (36).

This study also shows a single blast exposure to the head reduced the ASR but not sensorimotor gating as indicated by the lack of change in the PPI. ASR is a measure of the integrity of sensorimotor function and anxiety (23, 91), and a recent study reported decreased ASR following a non-blast TBI. In fact, the authors propose that suppression of ASR could be interpreted as an indication of reduced midbrain function since the neural circuitry for ASR involved auditory stimulation of the brainstem via the cochlear nucleus, caudal pontine nucleus, and reticulospinal tract for the spinal reflex that causes body movement (92). The suppressed ASR we detected was not accompanied by a PPI, which indicates that the sensorimotor gating function of the 80 psi head blast rats is intact. These findings suggest that suppressed ASR is a consequence of blast exposure and the lack of changes in PPI in blast TBI rats compared to sham indicates that at 2 weeks post-blast the auditory function is intact. Altogether, ASR and EPM studies show that a single blast exposure to the head results in depressed ASR, transient anxiety, and hyperarousal of defense responses, which are consistent with symptoms of GADs reported by service members exposed to blast TBI (21, 22).

The brain is vulnerable in the early stages post-TBI, making early diagnosis very crucial in preventing blast-exposed individuals from exposure to subsequent TBIs (blast/non-blast and sub-minimal TBI), and to facilitate the recovery and rehabilitation process (31, 93). This study provides evidence that a hypermetabolic state in the brain is evident 1 day post-blast (Figure 4) and that ^18^F-FDG PET imaging in the acute phase following injury (24 h) may be one means of early detection of TBI. Furthermore, this study confirms that a single blast loading injury from overpressure waves directed to the head (in the absence of peripheral contributions) is sufficient to produce a transient increase in overall cerebral glucose metabolism, increased anxiety-like and hyperarousal behaviors, and regional increases in markers of neuronal damage and astrogliosis. Our data also indicate that although learning memory was transiently affected in the first week post-blast (36), working memory was not intact until 2 weeks after the blast injury (Figure 2). This is consistent with findings that U.S. service members suffering from blast TBI more frequently display explicit attention deficits in working memory than deficits in learning memory (7, 54, 94, 95). Early intervention to prevent this early metabolic crisis as suggested to be beneficial in non-blast TBI (96) also would be of benefit in blast TBI.

## Author Contributions

HA, KS, and LG wrote the main manuscript text and prepared all figures. HA, LG, and ML performed experiments. HA and PT performed blast injuries. DB, LG, and VA provided expertise, equipment, and data analysis, especially regarding behavioral experiments (LG) and PET imaging (VA).

## Conflict of Interest Statement

The authors declare that the research was conducted in the absence of any commercial or financial relationships that could be construed as a potential conflict of interest.

## Supplementary Material

The Supplementary Material for this article can be found online at http://journal.frontiersin.org/article/10.3389/fneur.2015.00132
