# Supplementary material for: Blast Overpressure Waves Induce Transient Anxiety and Regional Changes in Cerebral Glucose Metabolism and Delayed Hyperarousal in Rats
Source: Front Neurol. 2015 Jun 17;6:132. doi: 10.3389/fneur.2015.00132 (PMC4470265; doi:10.3389/fneur.2015.00132)
Supplement: Supplementary file 1 [file Data_Sheet_1.DOCX]

**SUPPLEMENTARY DATA**

**Blast overpressure waves induce anxiety and transient regional changes in cerebral glucose metabolism in rats**

**Hibah O. Awwad^1,2^*, Larry P. Gonzalez^2,3^, Paul Tompkins^4^, Megan Lerner^5,7^, Daniel J. Brackett^5^, Vibhudutta Awasthi^1^, Kelly M. Standifer^1,2,6^**

^1^Department of Pharmaceutical Sciences, College of Pharmacy, ^2^Oklahoma Center for Neuroscience, ^3^Department of Psychiatry & Behavioral Sciences, ^4^Department of Neurosurgery, ^5^Department of Surgery and ^6^Department of Cell Biology, College of Medicine, University of Oklahoma Health Sciences Center; ^7^Oklahoma city VA Medical Center, Oklahoma City, OK, USA

*** Correspondence:** Corresponding Author:

Hibah O. Awwad, Department of Pharmaceutical Sciences

College of Pharmacy, University of Oklahoma Health Sciences Center,

1110 N. Stonewall Avenue, Suite 315, Oklahoma City, OK 73117, U.S.A.

[hawwad@ouhsc.edu](mailto:hawwad@ouhsc.edu)

**Number of figures and tables:** 1 supplementary figure and 1 supplementary table

**
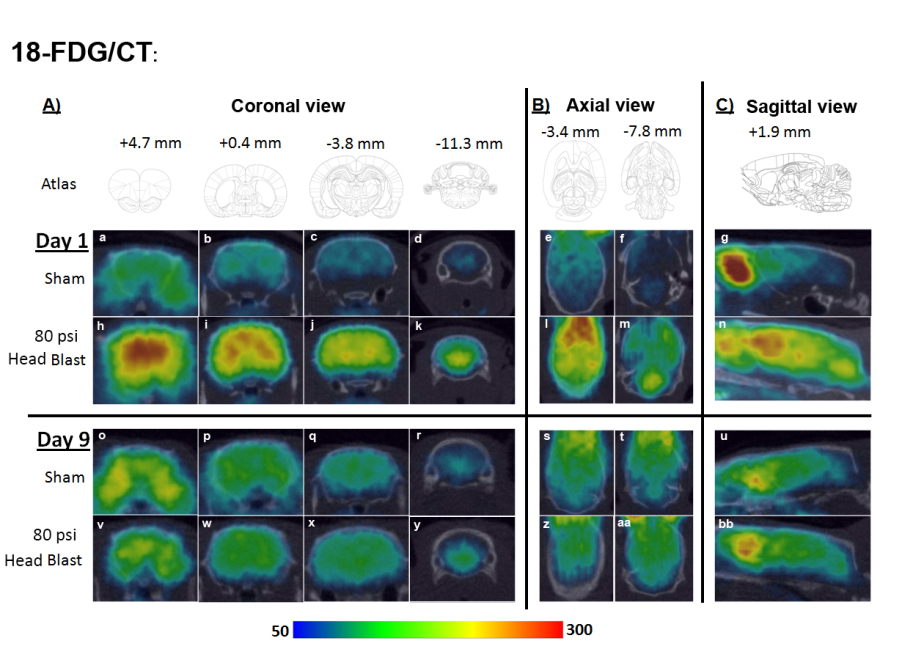
**

**Supplementary Fig S1. Three dimensional views representing glucose uptake in sham and blast rat brains on day 1 and day 9 post-blast.** Reconstructed PET images were fused with CT images using Amira Software as explained in methods. Representative images in coronal (A), axial (B) and sagittal (C) views from one rat per group of ^18^F-FDG uptake within the brain of sham (a-g; o-u) and blast (h-n; v-bb) rats on day 1 (a-n) and day 9 (o-bb) post-blast. Images are aligned below the corresponding rat brain atlas map (Brain Navigator) as indicated by the position of the section with reference to the Bregma. The color map spectrum indicates the intensity of ^18^F-FDG uptake, with red being the most intense and blue the least intense.

**Supplementary Table 1.** Blast wave parameters based on simulations of 80 psi from a similar blast device apparatus using the conventional weapons effects software ConWep 2.1.0.8, courtesy of Dr. Namas Chandra and Aaron Alai, University of Nebraska-Lincoln.

**Supplementary Table 1**

| **Aboveground Airblast, Spherical Free-Air Burst simulation with Conwep software** | |
| --- | --- |
| Equivalent weight of TNT | 0.384 pounds |
| Peak incident overpressure | 80 psi |
| Range to target | 2.315 feet |
| Normally reflected pressure | 364.5 psi |
| Time of arrival | 0.4609 msec |
| Positive phase duration | 0.9819 msec |
| Incident impulse | 11.49 psi/msec |
| Reflected impulse | 33.52 psi/msec |
| Shock front velocity | 1806 miles/hr |
| Peak dynamic pressure | 88.76 psi |
| Peak particle velocity | 1246 miles/hr |
| Shock density | 0.2463 lb/cubic foot |
| Specific heat ratio | 1.395 lb/cubic foot |
